# Electronic medical record-generated data use for decision-making and associated factors among healthcare managers in Somali public health facilities: a multicenter cross-sectional study

**DOI:** 10.3389/fdgth.2026.1851686

**Published:** 2026-06-19

**Authors:** Nor Haji Osman, Abdisalan Mohamed Roble, Abdirahman Mohamed Hussein, Abdiweli Mohamed Abdi, Liban Abdiwali Sheikh Ali, Aweis Ahmed Moallim, Ismail Dahir Ahmed, Abdullahi Mohamud Abdi, Abdirahman Mohamed Jimale, Abdikarim Abdi Adam, Dahir Mohamed Osman, Abdikarim Ali Omar, Mohamed Ahmed Omar, Marian Muse Osman, Tigad Abdisad Ali

**Affiliations:** 1 Center for Graduate Studies, Department of Statistics and Data Analytics, Jamhuriya University of Science & Technology (JUST), Mogadishu, Somalia; 2 Department of Policy and Planning, HIS Section, Directorate of Health and Human Services, Mogadishu, Banadir Region, Somalia; 3 Department of Digital Health and Innovation, HIS Section, Ministry of Health and Human Services, Mogadishu, Somalia; 4 Department of Environmental Health and Climate, IPC Section, Federal Ministry of Health and Human Services, Mogadishu, Somalia; 5 Department of Policy and Planning, HIS Section, Ministry of Health and Human Services, Baidoa, Southwest State, Somalia; 6 Research Department, Somali National Institute of Health, Mogadishu, Somalia; 7 Department of Infection Prevention and Control, Mogadishu Somali-Türkiye Recep Tayyip Erdoğan Training and Research Hospital, Mogadishu, Somalia

**Keywords:** data use, decision-making, digital health, electronic medical records, health managers, Somalia

## Abstract

**Background:**

Electronic medical record (EMR) systems have been introduced in public health facilities across Somalia to strengthen routine health information systems and support data-informed decision-making. However, evidence on the extent to which EMR-generated data are used for managerial and clinical decision-making remains limited. This study assessed EMR-generated data utilization and associated factors among healthcare managers in public health facilities in Somalia.

**Methods:**

A facility-based cross-sectional study was conducted from November to December 2025 across 45 public health facilities. A total of 405 healthcare managers were selected using a multistage cluster sampling technique. Data were collected using self-administered questionnaires and observational checklists developed based on the PRISM framework. The tool assessed organizational, technical, and behavioral factors influencing EMR data use. Data were entered and cleaned in Excel and analyzed using SPSS version 27. Descriptive statistics were used, and bivariable and multivariable binary logistic regression analyses identified factors associated with EMR-generated data utilization.

**Results:**

Overall, 145 healthcare managers (35.8%) reported adequate use of EMR-generated data for decision-making. EMR data were most commonly used to assess clinical guideline adherence (29.9%) and provider performance (28.4%), but least used for quality improvement (15.1%). Diploma-level qualification (AOR = 0.49; 95% CI: 0.25–0.95) and ≤1 year of administrative experience (AOR = 0.57; 95% CI: 0.36–0.90) were associated with lower EMR data use. Laboratory professionals were more likely to use EMR data than general practitioners (AOR = 2.96; 95% CI: 1.07–8.16). Creating performance dashboards (AOR = 2.25; 95% CI: 1.19–4.24), identifying patient care gaps (AOR = 5.02; 95% CI: 2.42–10.44), and access to technical support (AOR = 1.71; 95% CI: 1.02–2.89) were positively associated with EMR data use, while perceived system difficulty was negatively associated with utilization (AOR = 0.39; 95% CI: 0.22–0.70).

**Conclusion:**

EMR-generated data use for decision-making remains limited in Somalia's public health facilities. Strengthening user capacity, improving system usability, and ensuring technical support are essential to promote routine data-driven decision-making.

## Introduction

Electronic medical record (EMR) systems are designed to support evidence-based clinical and management decision-making while improving the accuracy, accessibility, and continuity of patient data (1–3). By enabling the routine use of digital data in healthcare settings, EMR systems can improve service quality, strengthen patient safety, and support performance monitoring, in addition to maintaining patient records (4–6). However, in many low- and middle-income countries (LMICs), routinely generated electronic data remain underutilized in decision-making processes, and EMR implementation has not consistently translated into meaningful data use (7–9). Similarly, a systematic review introducing the Staging and Barrier in EMR Adoption (SBEA) model highlights that EMR implementation is often constrained by both technical and human-factor barriers, helping to explain why electronic systems may remain primarily data-entry tools rather than mature decision-support systems (10). Limited utilization of EMR data has been linked to inadequate feedback systems, missed opportunities for quality improvement, inefficient resource allocation, and poor service planning (8).

Previous studies in the Banadir region of Somalia have highlighted broader routine health information system (RHIS)-related challenges, including low routine data utilization and poor data quality, despite the deployment of digital health tools such as the RAAD-EMR platform (11, 12). These studies identified critical determinants such as insufficient training, lack of standardized indicators, and inadequate supervision, all of which hinder the effective use of health data for decision-making.

Evidence from Sub-Saharan Africa suggests that EMR adoption is frequently characterized by inconsistent system functionality, limited user readiness, parallel paper-based and electronic processes, and limited coverage (9). Despite investments in digital health platforms, studies from Tanzania (40.6%) (1), Eastern Ethiopia (26.6%) (2), and the Amhara region of Ethiopia (45.3%) (13) have shown similarly low levels of EMR data use for routine decision-making. Recent evidence from India also shows that data-informed decision-making among sub-district public health administrators remains an emerging area of empirical inquiry, with quantifying the Data Utilization Score among medical officers at primary health centres and highlighting the need to strengthen routine data use at the service-delivery level (14). Earlier research has also shown that methods for querying patient data, assessing adherence to clinical recommendations, and identifying treatment gaps among specific patient populations were among the least commonly developed uses of EHR data. Although EHRs are used by a wide range of health organizations and care providers, hospital characteristics, such as public or private ownership and rural or urban location, significantly influence how EMR data are used. Considerable variation also exists in the extent to which electronic data support decision-making (15).

In many settings, health data are collected but remain unanalyzed and underutilized, limiting their contribution to policy and program improvement (8, 9, 16). Commonly reported constraints include inadequate training, limited technical support, weak organizational data-use culture, insufficient data-analysis skills, and perceptions of poor system usability (2, 13, 17). While these studies provide valuable insights into EMR adoption and functionality, most have focused primarily on system deployment and adoption indicators, with fewer studies examining how EMR-generated data are actually used to inform clinical, administrative, or managerial decisions at the facility level (9).

Recent national digital health initiatives in Somalia have supported the implementation of the RAAD EMR platform in selected public health facilities, contributing to the strengthening of routine health information systems (18). RAAD is an adapted, open-source electronic medical record platform built on Bahmni/OpenMRS for low-resource settings. It is designed to support patient registration, clinical documentation, prescriptions, laboratory results, and routine reporting in Somali public health facilities (18). However, EMR utilization remains inconsistent across institutions, parallel paper-based and electronic systems persist, and routine electronic data are not consistently transformed into actionable insights for decision-making (19, 20). The current literature in Somalia predominantly focuses on infrastructure development, implementation experiences, and health information system preparedness, with limited empirical evidence on the utilization of EMR-generated data by healthcare managers for operational planning, service monitoring, or performance improvement (19, 20). To date, no previous study in Somalia has systematically examined the extent to which EMR-generated data inform healthcare managers’ decision-making or explored the individual, organizational, behavioral, and technical factors associated with EMR data utilization.

The Ministry of Health and Human Services in Somalia has prioritized digital health as part of broader efforts to strengthen the health system. These efforts include investments in routine health information systems, interoperability initiatives, and the integration of electronic medical record platforms such as RAAD with national data systems (21). Effective utilization of EMR-generated data is crucial for assessing service delivery performance, identifying gaps in patient care, guiding resource allocation, and supporting quality improvement programs at both facility and system levels. Without regular analytical use, EMR systems are likely to function mainly as data-entry tools rather than decision-support systems, thereby limiting their potential to improve health system efficiency and patient outcomes (22).

Furthermore, previous research in similar LMIC settings has not adequately integrated individual, organizational, behavioral, and technical factors when analyzing the determinants of EMR data utilization. Studies have been descriptive, focusing on availability or acceptance rather than actual use, while others have not applied structured analytical frameworks to explain routine data-use behavior (2, 3). Evidence from public healthcare centers in Haryana, India, also shows that data use for programmatic evidence-based decision-making is shaped by multiple determinants at the service-delivery level. This reinforces the need to examine organizational, technical, and behavioral factors that influence routine data use in LMIC health systems (23). The Performance of Routine Information System Management (PRISM) framework provides a comprehensive approach to understanding how technical capacity, organizational support, and user behavior influence the use of routine health information for decision-making. Applying this framework can help identify actionable factors to strengthen data-use culture and improve the effectiveness of digital health investments (24–26).

This study addresses these gaps by examining the extent of EMR data use in decision-making and identifying associated individual, organizational, behavioral, and technical factors among healthcare managers in public health facilities in the Banadir and Bay Regions of Somalia. The study applies the PRISM framework (15) to generate context-specific evidence that can inform strategies to strengthen routine data use, enhance digital health implementation, and improve data-driven decision-making within Somalia's health system.

## Methods and materials

### Study design and setting

This facility-based cross-sectional study employed a quantitative approach and was conducted from November to December 2025. The study was conducted in selected public health facilities with functional RAAD-EMR systems in Banadir Region and Bay Region, Southwest State, Somalia. These two regions were selected using a simple random sampling method from regions where RAAD-EMR had been implemented. The study included 45 public health facilities, comprising 41 health centers and 4 hospitals. Somalia is situated in the Horn of Africa and comprises 18 administrative regions. According to the 2014 Population Estimation Survey, Somalia's population was estimated at 12.3 million, with approximately three-quarters of the population below 30 years of age (27, 28). The national health facility network comprises 1,219 facilities. These included health centers, primary health units, private clinics, district hospitals, regional hospitals, national referral hospitals, specialty hospitals, TB centers (29). At the time of the study, roughly 70 public primary health facilities had implemented and were utilizing the RAAD-EMR system. These facilities formed the sampling frame.

RAAD is an Electronic Medical Record (EMR) platform utilized in Somalia as an open-source digital health instrument for the management of patient data and the improvement of healthcare service delivery (18). RAAD, built on the Bahmni system, facilitates effective digital management of patient data and optimizes clinical operations. The platform aims to enhance data quality, patient care, and clinical decision-making in healthcare institutions across Somalia. RAAD is being implemented in collaboration with Somalia's Federal Ministry of Health and Federal Member State partners as part of ongoing efforts to strengthen the country's digital health infrastructure (30). Under the Health Alliance for Digital Development and Action (HADDA) initiative, RAAD has been piloted and scaled in selected public health facilities, particularly in Banadir, Puntland, and Galmudug, with evidence suggesting expansion to approximately 30 facilities and preparation for further rollout. The system supports patient registration, clinical documentation, prescriptions, laboratory results management, reporting, and integration with Somalia's national health information system, including DHIS2. By promoting interoperability, reducing reliance on paper-based records, and improving the availability of timely and reliable patient-level data, RAAD contributes to data-informed clinical and administrative decision-making and supports Somalia's broader transition toward a more coordinated digital health ecosystem (31).

### Study population

The study population comprised healthcare managers and departmental/unit heads responsible for clinical and/or administrative decision-making in selected public health facilities with functional RAAD-EMR systems. Eligible participants were individuals aged ≥18 years working in facilities with functional RAAD-EMR systems. Participants who did not provide consent, worked in facilities using program-specific EMR platforms (e.g., Ogow, Caafimaad Plus) rather than RAAD, or were on leave during data collection were excluded.

### Sample size determination

The sample size was determined using the single population proportion formula. In the absence of prior estimates, a proportion of 50% (*P* = 0.50) was assumed, with a 95% confidence level and a 5% margin of error. To account for potential non-response, an additional 5% was included. Based on these parameters, the sample size was calculated using the formula:

$$n=\frac{Z^{2}*p(1-p)}{d^{2}}n=\frac{{\left(1.96\right)}^{2}*0.5(1-0.5)}{(0.05^{)2}}n=\frac{3.8416*0.25}{0.0025}n=384.16$$

n_final_= 384 + non-response rate (5%) is equal to 405. Therefore, the final sample size used in this study was 405 participants.

### Sampling procedure

**Figure 1:**
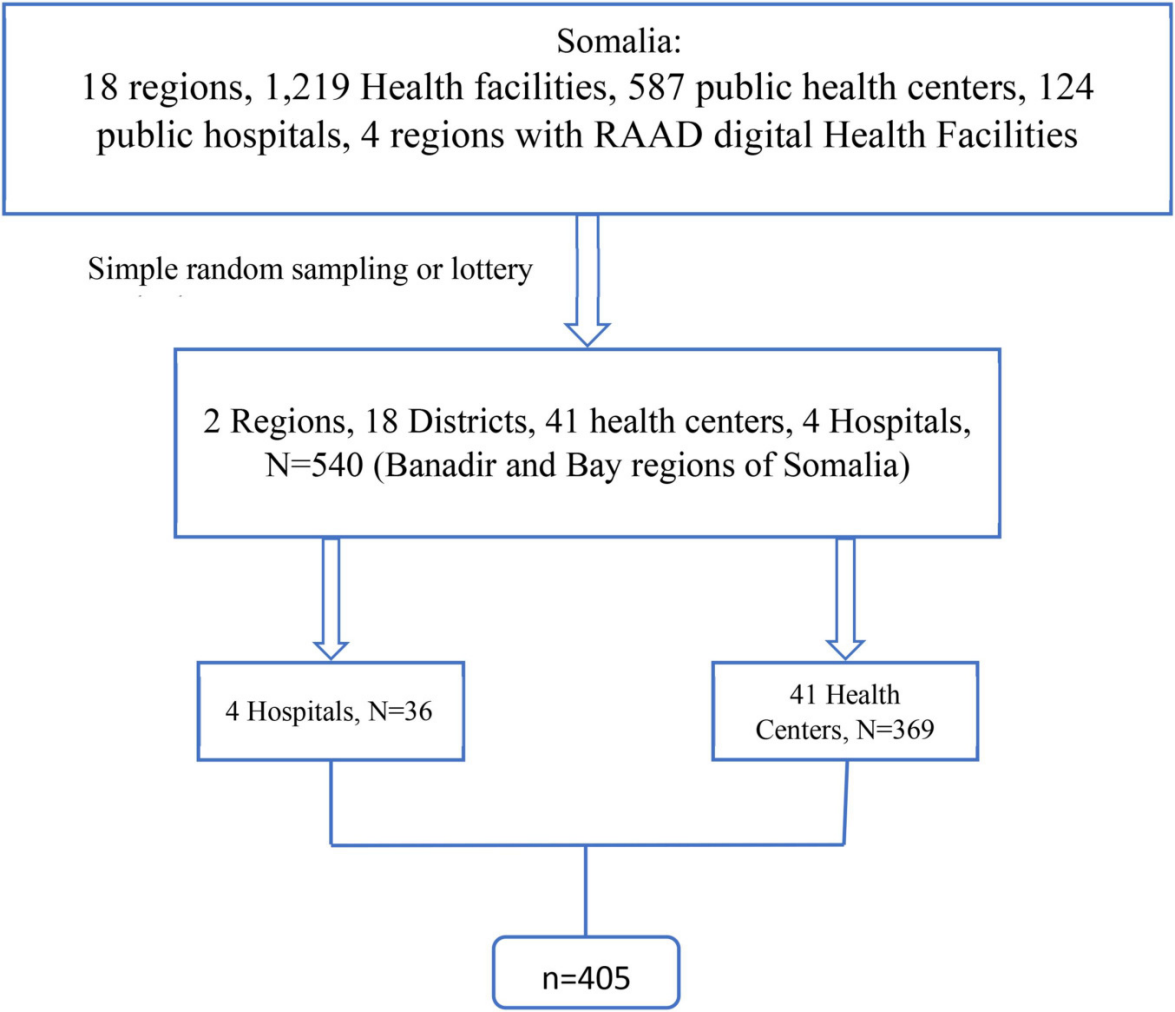
Schematic presentation of the sampling procedure for EMR system generated data use in Somalia (*n* = 405).

A multistage sampling approach was used, in which two regions were selected by simple random (lottery) sampling, followed by stratified sampling of hospitals and health centers with proportionate allocation to obtain the final sample size (*n* = 405) (Figure 1).

### Data collection methods and data collection procedure

Data were collected using a structured self-administered questionnaire supplemented by a documentary review checklist. These tools were adapted and constructed from PRISM tools (26, 32) and from previously conducted studies (1, 2, 33). It consists of sociodemographic, organizational, behavioral, technical, knowledge, and attitude-related variables. The questionnaire was developed in English using Kobo Toolbox, translated into Somali, and back-translated to ensure conceptual consistency. A pretest was conducted in a facility outside the study area using 12.5% of the sample (*n* = 51), and revisions were made to improve clarity and sequencing.

Document observation covered EMR data-use practices over the preceding six months, including HMT meeting minutes, commodity audit reports, and procurement records. Data were collected through facility visits by five trained data collectors and two HIS supervisors from non-study districts to reduce bias. All field staff received two days of training on study procedures, ethical conduct, and data quality assurance.

Questionnaires were completed privately after obtaining informed consent, while document review and limited observation were used to validate self-reported responses. Supervisors conducted daily data checks to ensure completeness and consistency, and feedback was provided immediately when discrepancies were identified. Prior to analysis, datasets were cleaned, verified, and assessed for statistical assumptions.

### Variable measurements

#### Knowledge of computer and data use

Knowledge of computer and data use was measured using a 10-item competency scale covering: internet browsing, performing calculations, email communication, EMR database management, data accuracy checking, plotting data by month or year, computing trends from bar charts, interpreting findings, using data to identify gaps and set targets, and applying data for decision-making and feedback. Scores were categorized as follows: ≥90 = excellent, 80–<90 = very good, 60–<80 = good, 50–<60 = fair, and ≤50 = poor (1, 2).

#### Attitude

Attitude toward EMR use was assessed using eight Likert-scale items (strongly disagree to strongly agree). Participants with a total score above 32 were classified as having a positive (good) attitude, whereas those scoring below 32 were categorized as having a negative (poor) attitude (1, 2).

#### Perceived EMR generated data system quality

Perceived EMR system quality was assessed using five Likert-scale items (strongly disagree to strongly agree). Participants with a total score ≥20 were classified as having good perceived system quality, whereas those scoring <20 were categorized as having poor perceived system quality (1, 2).

#### Perceived EMR generated data service quality

Perceived EMR service quality was assessed using nine Likert-scale items (strongly disagree to strongly agree). Participants with a total score ≥36 were classified as having good perceived service quality, whereas those scoring <36 were categorized as having poor perceived service quality.

#### Perceived EMR generated information quality

Perceived EMR information quality was assessed using seven Likert-scale items (strongly disagree to strongly agree). Participants with a total score ≥28 were classified as having good perceived information quality, whereas those scoring <28 were categorized as having poor perceived information quality (1, 2).

#### EMR generated data utilization in decision-making (health manager)

EMR data use in decision-making was defined as the use of EMR-generated data to support clinical or managerial decisions. The extent of data use was assessed using a 10-item scale covering quality improvement, clinical guideline adherence, performance dashboards, provider profiling, identification of high-risk patients and care gaps, strategic planning reports, unit performance monitoring, and data-query functions. Participants with a mean score >5 were classified as having adequate EMR data use, whereas scores ≤5 indicated inadequate data use (10).

### Data analysis

The collected data were cleaned in Excel and then exported to SPSS version 27 for coding and statistical analysis. Descriptive statistics (frequencies, percentages) were used to summarize participant characteristics and key study variables, and findings were presented using tables and figures.

The outcome variable EMR data use in decision-making was dichotomized as adequate vs. inadequate use. Two levels of analysis were conducted. First, descriptive analysis was performed to determine the proportion of respondents and facilities reporting EMR data use. Second, bivariable logistic regression was used to assess the association between EMR data use and individual, organizational, behavioral, technical, and demographic factors. Variables with a *p*-value <0.25 in the bivariable analysis were included in the multivariable logistic regression model to control for potential confounding. Independent predictors were reported using adjusted odds ratios (AORs) with 95% confidence intervals (CIs), and statistical significance was declared at *p* < 0.05.

All statistical assumptions were checked prior to final modeling. Multicollinearity was assessed using the Variance Inflation Factor (VIF), applying a cutoff value of 6, and no evidence of multicollinearity was detected. Model fitness was assessed using the Hosmer–Lemeshow goodness-of-fit test.

## Results

### Sociodemographic characteristics

A total of 405 healthcare managers participated in the study, with 225 (55.6%) from Banadir Region and 180 (44.4%) from Southwest (Bay Region). The majority of respondents (91.1%) were employed at health centers. More than half were female (55.3%) and under 30 years of age (53.3%). Most participants held a degree or higher qualification (83.7%), while 16.3% possessed a diploma. Nurses represented the largest professional group (36.3%), followed by nutritionists (18.0%), midwives (17.3%), general practitioners (11.4%), laboratory professionals (11.1%), and pharmacists (5.9%). In total, 53.1% had more than six years of work experience, and 57.3% reported over one year of administrative experience (Table 1).

**Table 1:** Sociodemographic characteristics of the study participants.

Variables	Category	Frequency	Percentage
Region	Banadir region	225	55.56
	Southwest (Bay region)	180	44.44
Health Facility Type	Health Center	369	91.11
	Hospital	36	8.89
Sex	Female	224	55.3
	Male	181	44.7
Age (Years)	<30 years	216	53.3
	>30 years	189	46.7
Highest Education level	Degree and above	339	83.7
	Diploma	66	16.3
Professional cadre	General Practitioners	46	11.4
	Medical laboratory	45	11.1
	Midwives	70	17.3
	Nurse	147	36.3
	Nutrition	73	18.0
	Pharmacists	24	5.9
Current position	EPI Incharge	61	15.1
	Facility Incharge	52	12.8
	Facility Screening	23	5.7
	HMIS focal person	45	11.1
	Laboratory Incharge	20	4.9
	Maternity Incharge	66	16.3
	OPD Incharge	101	24.9
	Pharmacy Incharge	37	9.1
Work experience	<6 years	190	46.9
	>6 years	215	53.1
Years of administrative experience	>1 year	232	57.3
	≤1 year	173	42.7

### EMR generated data utilization in decision-making among healthcare managers, Somalia, 2025

Regarding EMR-related practices, 39 (9.6%) participants reported previous EMR use, while 113 (27.9%) reported current EMR use in their facility. Among current EMR users, 51 (45.1%) reported using EMR daily, 32 (28.4%) reported using it three times per week, 12 (10.6%) reported using it once per week, and 18 (15.9%) could not recall the exact frequency of use. Overall, 49 (12.1%) respondents directly reported using EMR data for decision-making based on a single-item question. However, based on the composite 10-item EMR data-use scale, 145 (35.8%) participants were classified as having adequate EMR data-use practices, while 260 (64.2%) had inadequate data-use practices (Table 2).

**Table 2:** EMR system data use practices in decision-making among healthcare managers, Somalia, 2025 (*n* = 405).

Variables	Category	Frequency	Percentage
Past Electronic Medical Record (EMR) use	No	366	90.4
	Yes	39	9.6
Current EMR use	No	292	72.1
	Yes	113	27.9
Frequency of EMR use among current EMR users, *n* = 113	Daily	51	45.1
	Could not recall exact frequency	18	15.9
	Once a week	12	10.6
	Three times a week	32	28.4
EMR data use	No	356	87.9
	Yes	49	12.1
Extent of data use	Adequate data use	145	35.8
	Inadequate data use	260	64.2

Footnote: Percentages were calculated using the total sample size of 405, except for frequency of EMR use, which was calculated among current EMR users only (*n* = 113).

**Figure 2:**
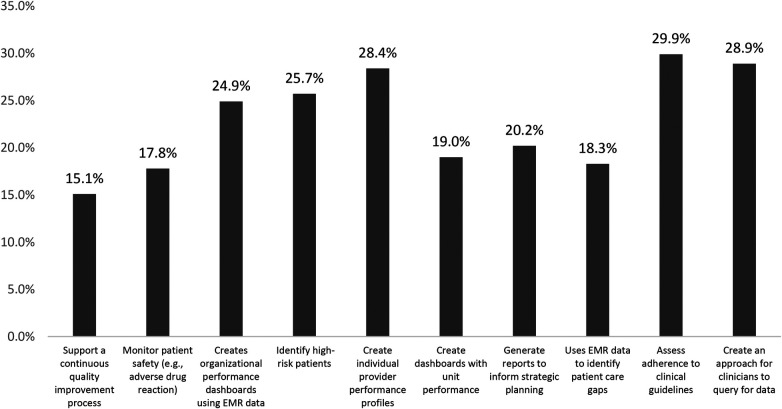
Purpose of EMR use by health facility managers in public health facilities, Somalia, 2025 (*n* = 405).

EMR data were most commonly used to assess adherence to clinical guidelines (29.9%), create individual provider performance profiles (28.4%), and support clinician data-querying approaches (28.9%). Other reported uses included identifying high-risk patients (25.7%), creating organizational performance dashboards (24.9%), generating strategic planning reports (20.2%), and unit performance dashboards (19.0%). The least frequently reported application was using EMR to identify patient care gaps (18.3%) and supporting continuous quality improvement processes (15.1%) (Figure 2).

**Figure 3:**
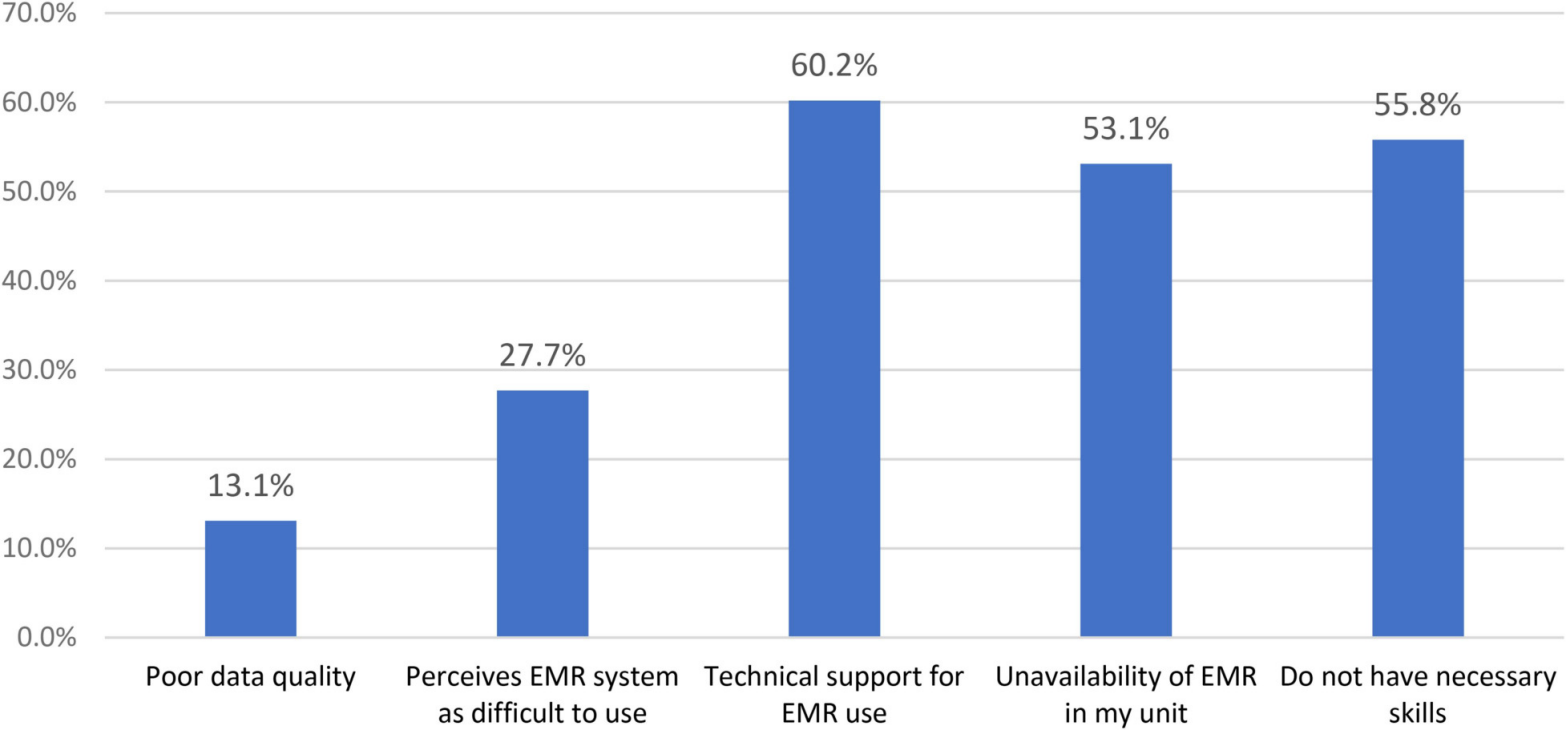
Common reasons for the frequent non-use of EMR data in decision-making, Somalia, 2025 (*n* = 405).

Regarding barriers to frequent EMR data use, participants reported lack of necessary skills (55.8%), unavailability of EMR in some units (53.1%), and limited technical support (60.2%) as the most common constraints. Additionally, 27.7% perceived the EMR system as difficult to use and 13.1% reported poor data quality as a barrier (Figure 3).

### EMR system data use and related organizational characteristics

Organizational and system-related characteristics were evaluated. Most respondents indicated that a computer was available in their unit (367; 90.6%), although the majority of facilities had only 1–3 accessible computers (270; 66.7%). Over two-thirds reported that available computers were functional (287; 70.9%) and shared among staff (286; 70.6%). A total of 291 participants (71.9%) had received training on the RAAD-EMR system, while 168 (41.5%) reported that EMR-related discussions occurred within their facilities. Only 85 (21.0%) reported motivation or incentives to encourage EMR use. Additionally, 287 respondents (70.9%) had previously received HMIS training, but only 54 (13.3%) reported the availability of designated EMR champions (Table 3).

**Table 3:** Organizational factors and EMR system data use, Somalia (*n* = 405).

Variables	Category	Frequency	Percentage
Availability of computer	No	38	9.4
	Yes	367	90.6
Number of accessible computers	1–3 Computers	270	66.7
	4–6 Computers	36	8.9
	7 and above Computers	99	24.4
Functionality of computer	No	118	29.1
	Yes	287	70.9
Sharing computers	No	119	29.4
	Yes	286	70.6
Training on RAAD EMR	No	114	28.1
	Yes	291	71.9
Discussion on EMR	No	237	58.5
	Yes	168	41.5
Motivation on EMR use	No	320	79.0
	Yes	85	21.0
Trained on HMIS	No	118	29.1
	Yes	287	70.9
Availability of EMR champion	No	351	86.7
	Yes	54	13.3

### EMR system data use in decision-making and related behavioral characteristics

The study assessed preferences for the electronic medical record (EMR) system compared to the paper-based system, as well as attitudes and knowledge regarding computer and data use. Of the participants, 319 (78.8%) preferred the EMR system over the paper-based system. Knowledge of computers and data use ranged from 26.2% to 91.4% across the ten areas evaluated. Additionally, 289 (71.4%) of health managers demonstrated a positive attitude toward the EMR system (Table 4).

**Table 4:** EMR system data use in decision-making and related behavioral characteristics, Somalia (*n* = 405).

Variables	Category	Frequency	Percentage
Prefer EMR than paper-based system	No	86	21.2
	Yes	319	78.8
Attitude level	Good	289	71.4
	Poor	116	28.6
Knowledge of computer and data use			
1. Internet browsing	No	35	8.6
	Yes	370	91.4
2. Calculation	No	110	27.2
	Yes	295	72.8
3. Using Email	No	68	16.8
	Yes	337	83.2
4. EMR database management	No	302	74.6
	Yes	103	25.4
5. I can check data accuracy	No	106	26.2
	Yes	299	73.8
6. I can plot data by months or years	No	114	28.2
	Yes	291	71.9
7. I can compute trends from bar charts	No	115	28.4
	Yes	290	71.6
8. I can explain findings and their implication	No	80	19.8
	Yes	325	80.3
9. I can use data for identifying gaps and setting targets	No	150	37.0
	Yes	255	63.0
10. I can use data for making various types of decisions and providing feedback	No	167	41.2
	Yes	238	58.8

### EMR system data use and related technical characteristics

The majority of participants reported basic computer skills, with 332 (82.0%) indicating computer proficiency. Among system perception measures, 153 (37.8%) of respondents reported a positive perception of EMR system quality, while 352 (86.9%) indicated good perceived EMR service quality. Additionally, 238 (58.8%) participants reported a favorable perception of EMR information quality (Table 5).

**Table 5:** EMR system data use and related technical characteristics, Somalia (*n* = 405).

Variables	Category	Frequency	Percentage
Ability to use computer	No	73	18.0
	Yes	332	82.0
Perceived EMR system quality	Good	153	37.8
	Poor	252	62.2
Perceived EMR service quality	Good	352	86.9
	Poor	53	13.1
Perceived EMR information quality	Good	238	58.8
	Poor	167	41.2

### Factors associated with EMR data use among health managers, Somalia (*n* = 405)

Overall model assessment showed that the final multivariable logistic regression model was statistically significant based on the Omnibus Test of Model Coefficients, *χ*^2^(6) = 67.459, *p* < 0.001. The Cox and Snell pseudo-R^2^ and Nagelkerke pseudo-R^2^ values were 0.153 and 0.211, respectively, indicating modest explanatory power. The Hosmer–Lemeshow goodness-of-fit test was not statistically significant, *χ*^2^(8) = 6.147, *p* = 0.631, suggesting no evidence of poor model fit. The constant/intercept was statistically significant, B = −3.059, *p* = 0.002.

Healthcare workers holding a diploma had lower odds of adequate use of electronic medical records (EMRs) than those with a degree or higher [AOR = 0.49; 95% CI: 0.25–0.95; *p* = 0.034]. Medical laboratory professionals exhibited significantly higher odds of adequate EMR data use than general practitioners [AOR = 2.96; 95% CI: 1.07–8.16; *p* = 0.037]. No significant associations were identified among nurses, midwives, nutritionists, or pharmacists. Participants with one year or less of administrative experience had reduced odds of using EMR data compared with those with greater experience [AOR = 0.57; 95% CI: 0.36–0.90; *p* = 0.017]. Healthcare workers who reported creating organizational performance dashboards [AOR = 2.25; 95% CI: 1.19–4.24; *p* = 0.013] and those who used EMRs to identify patient care gaps [AOR = 5.02; 95% CI: 2.42–10.44; *p* < 0.001] had higher odds of adequate EMR use. In contrast, perceiving the EMR as difficult to use was associated with reduced odds of EMR utilization [AOR = 0.39; 95% CI: 0.22–0.70; *p* < 0.001]. Access to technical support was positively associated with adequate EMR data use [AOR = 1.71; 95% CI: 1.02–2.89; *p* = 0.044]. Other variables did not reach statistical significance in the adjusted model (Table 6).

**Table 6:** Factors associated with EMR data use among health managers, Somalia (*n* = 405).

Variables	Level of EMR data use		COR (95% CI)	AOR (95% CI)	*p*-value
	Inadequate, *n* (%)	adequate, *n* (%)			
Highest educational qualification					
Degree and above	212 (62.5)	127 (37.5)	1^Reference^	1^Reference^	
Diploma	48 (72.7)	18 (27.3)	0.626 (0.349–1.123)	0.487 (0.250- 0.949)	0.034
Professional cadre					
General Practitioners	35 (76.1)	11 (23.9)	1^Reference^	1^Reference^	
Medical laboratory	25 (55.6)	20 (44.4)	2.545 (1.038–6.242)	2.955 (1.070–8.160)	0.037
Midwives	48 (68.6)	22 (31.4)	1.458 (0.627- 3.394)	1.072 (0.399–2.881)	0.891
Nurse	87 (59.2)	60 (40.8)	2.194 (1.033–4.660)	1.986 (0.856–4.605)	0.110
Nutrition	51 (69.9)	22 (30.1)	1.373 (0.591–3.186)	1.387 (0.522–3.685)	0.511
Pharmacists	14 (58.3)	10 (41.7)	2.273 (0.790–6.541)	2.769 (0.826–9.282)	0.099
Years of administrative experience					
>1 year	134 (57.8)	98 (42.2)	1^Reference^	1^Reference^	
≤1 year	126 (72.8)	47 (27.2)	0.510 (0.334–0.780)	0.566 (0.355–0.902)	0.017
Creates organizational performance dashboards using EMR data					
No	222 (73.0)	82 (27.0)	1^Reference^	1^Reference^	
Yes	38 (37.6)	63 (62.4)	5.256 (3.081–8.965)	2.246 (1.190–4.240)	0.013
Uses EMR data to identify patient care gaps					
No	240 (72.5)	91 (27.5)	1^Reference^	1^Reference^	
Yes	20 (27.0)	54 (73.0)	7.121 (4.039–12.553)	5.020 (2.415–10.435)	<0.001
Perceives EMR system as difficult to use					
No	178 (60.8)	115 (39.2)	1^Reference^	1^Reference^	
Yes	82 (73.2)	30 (26.8)	0.566 (0.351–0.915)	0.391 (0.219–0.696)	<0.001
Technical support for EMR use					
No	96 (59.6)	65 (40.4)	1^Reference^	1^Reference^	
Yes	164 (67.2)	80 (32.8)	0.720 (0.477–1.089)	1.713(1.015–2.892)	0.044

*P* value < 0.05 from multivariable analysis.

COR, crude odds ratio; AOR, adjusted odds ratio.

## Discussion

This study provides one of the first empirical assessments of the extent to which EMR-generated data are used for managerial and clinical decision-making within public health facilities in Somalia. Although EMR systems have been rolled out through the RAAD platform as part of national digital-health strengthening efforts, the findings indicate that meaningful data use remains limited, with only one-third 145 (35.8%) of health managers demonstrating adequate EMR data-use practices. This illustrates a persistent implementation–to–use gap, where digital systems are installed but routine data are not consistently transformed into actionable information for planning, performance monitoring, or service improvement. Similar gaps have been reported in Tanzania, Ethiopia, and Kenya, where EMR implementation did not automatically translate into strengthened decision-making or data-use culture despite substantial investments in digital health (1, 2, 9, 13). These regional patterns suggest that the presence of EMR infrastructure alone is insufficient to institutionalize data-driven decision-making in resource-constrained health systems.

Furthermore, the proportion of adequate EMR data use observed in this study is higher than that reported in Dire Dawa, Ethiopia (26.6%) (2), but comparable to findings from Amhara Region and Kenya, where partial system coverage, hybrid paper electronic workflows, and variable system readiness contributed to low levels of data utilization (7, 9, 13). The Somali context reflects these regional trends: EMR availability remains uneven across units, most facilities rely on shared computers, and only a minority of health managers reported engaging with EMR data for dashboards, care-gap analysis, or strategic planning. These findings align with evidence from Sub-Saharan Africa indicating that digitalization alone does not improve data-driven decision-making unless organizations simultaneously strengthen capacity, accountability mechanisms, and feedback structures that encourage active use of routine health information (8, 9, 16). Consequently, these findings reinforce growing evidence from Sub-Saharan Africa that digitalization alone does not improve data-driven decision-making unless organizations simultaneously strengthen workforce capacity, managerial accountability mechanisms, and feedback structures that promote routine use of health information.

From an individual-level perspective, educational status, professional cadre, and administrative experience were significantly associated with EMR data use. Health managers with diploma-level qualifications and those with ≤1 year of managerial experience had lower odds of adequate data use, suggesting that analytical competency, familiarity with supervisory responsibilities, and confidence in interpreting digital data are central to effective engagement with EMR platforms. In line with this interpretation, comparable findings from Ethiopia and Tanzania indicate that higher educational attainment and prior exposure to data management are important predictors of digital system use (1, 2, 5, 13). Moreover, laboratory professionals demonstrated higher odds of EMR data use than general practitioners, which may reflect a longstanding laboratory culture of structured reporting, data verification, and routine performance monitoring. This pattern supports prior evidence that professional roles, task expectations, and routine reporting obligations shape how different cadres interact with routine information systems (3, 6, 15). The higher odds of EMR data use among laboratory professionals may be explained by their routine involvement in structured test documentation, results verification, and reporting, which may promote stronger data-use practices compared with general practitioners. However, this finding should be interpreted cautiously because professional differences may also reflect workflow design, access to EMR functions, and facility-level implementation practices.

At the behavioral and organizational level, EMR data use was more likely among managers who reported generating organizational performance dashboards and identifying patient care gaps. These activities require deliberate interrogation of EMR datasets and therefore reflect a shift from passive data entry toward proactive and purposeful analytic use. Consistent with the Performance of Routine Information System Management (PRISM) framework, this finding suggests that data-use behavior improves when decision-makers perceive data as relevant, problem-solving tools for monitoring and quality improvement (15, 34). Nevertheless, the limited evidence of routine review meetings, weak feedback culture, and the absence of designated EMR “champions” indicates that institutional structures supporting data-use practices remain fragile, a constraint that has also been documented across other LMIC health systems (16, 17, 19). This implies that strengthening EMR use requires not only individual skill-building but also deliberate organizational strategies that embed data use within decision-making processes.

From a technical perspective, perceiving the EMR system as difficult to use was associated with a reduced likelihood of adequate data use, whereas access to technical support significantly increased EMR utilization. This aligns with prior research showing that usability barriers, intermittent system functionality, inadequate ICT support, and limited hands-on training undermine user engagement with digital platforms (1, 2, 17). Although most respondents reported computer availability and prior HMIS training, the dominance of shared workstations and hybrid workflows may fragment data continuity and discourage routine analytical use. In addition, the most frequently reported barriers, including lack of skills, limited technical support, and incomplete EMR coverage across units, mirror findings from other African digital-health implementations, where weak implementation support structures constrained the meaningful application of routine data (8, 9, 19). Collectively, these results highlight that technical readiness, user experience, and implementation support are critical determinants of whether EMR systems evolve from data-entry tools into decision-support instruments.

## Limitations

The study focused exclusively on healthcare managers working in public health facilities with functional RAAD-EMR systems. Therefore, the findings may not fully represent facilities without functional EMR systems, private facilities, or rural health facilities where digital health implementation may differ. The use of self-administered questionnaires may have introduced social desirability bias, as participants could have overreported their use of EMR-generated data for decision-making. In addition, the cross-sectional design limits causal interpretation and does not allow assessment of changes in EMR data-use practices over time. Although a multistage sampling approach was used, the sample size calculation did not apply a design effect, which may have affected the precision of the estimates. Therefore, the findings may be most generalizable to public health facilities in Somalia with functional RAAD-EMR systems and similar socio-demographic, organizational, and digital-health implementation contexts, rather than to all health facilities nationally. Future studies using nationally representative samples, appropriate design-effect adjustment, and longitudinal or mixed-methods designs are recommended to better understand barriers and facilitators of EMR data use across Somalia.

## Conclusion

This study shows that although EMR systems have been introduced in public health facilities in Somalia, the use of EMR-generated data for managerial and clinical decision-making remains limited, with only one-third of health managers demonstrating adequate data-use practices. EMR utilization was influenced by educational level, managerial experience, professional role, technical support, and perceived system usability, indicating that digital-system availability alone is insufficient to ensure meaningful data use. Strengthening capacity-building, institutionalizing routine data-review mechanisms, and improving technical support and system usability are essential to translate EMR implementation into effective, evidence-based decision-making and improved health-service performance.

## Data Availability

The raw data supporting the conclusions of this article will be made available by the authors, without undue reservation.
